# Supplementary material for: Evaluation of Clinical Parameters Associated with Response and Resistance to Cemiplimab in Locally Advanced and Metastatic Cutaneous Squamous Cell Carcinoma: A Multi-Institutional Retrospective Cohort Study
Source: Curr Oncol. 2025 Mar 15;32(3):168. doi: 10.3390/curroncol32030168 (PMC11941019; doi:10.3390/curroncol32030168)
Supplement: Supplementary file 1 [file curroncol-32-00168-s001.zip › curroncol-3443232-SI.pdf]

Table S1. details of immunosuppressive therapy for solid organ transplant recipients receiving cemiplimab therapy.

**Patient**	**Transplant organ**	**Immunosuppressive therapy prior to cemiplimab**	**Immunosuppressive therapy during cemiplimab**
1	Renal	Tacrolimus 2mg BD; Prednisolone 5mg OD	Continued until radiological evidence of progression, then began weaning off tacrolimus. Cemiplimab discontinued on further progression after weaning to tacrolimus 1mg BD.
2	Renal	Tacrolimus 1mg OD; Prednisolone 5mg/10mg alternate days	Continued throughout treatment with cemiplimab
3	Renal	Tacrolimus 2mg BD; Azathioprine 50mg alternate days	Continued throughout treatment with cemiplimab
